# Microbiota-Derived Short-Chain Fatty Acids Promote LAMTOR2-Mediated Immune Responses in Macrophages

**DOI:** 10.1128/mSystems.00587-20

**Published:** 2020-11-03

**Authors:** Ting Wu, Hongru Li, Cong Su, Fangming Xu, Guangwei Yang, Kaili Sun, Mengran Xu, Na Lv, Bao Meng, Yanyan Liu, Lifen Hu, Yan Liu, Yufeng Gao, Heng Wang, Yanhu Lan, Dexiang Xu, Jiabin Li

**Affiliations:** a Department of Infectious Diseases, The First Affiliated Hospital of Anhui Medical University, Hefei, Anhui, China; b XiangYa School of Medicine, Central South University, Changsha, Hunan, China; c Department of Infectious Diseases, The Chaohu Affiliated Hospital of Anhui Medical University, Hefei, Anhui, China; d Anhui Center for Surveillance of Bacterial Resistance, Hefei, Anhui, China; e Institute of Bacterial Resistance, Anhui Medical University, Hefei, Anhui, China; f Department of Basic Medical, Anhui Medical University, Hefei, Anhui, China; g Department of Hospital Management, The First Affiliated Hospital of Anhui Medical University, Hefei, Anhui, China; h Laboratory of Environmental Toxicology, Department of Toxicology, Anhui Medical University, Hefei, Anhui, China; University of California, San Francisco

**Keywords:** *K. pneumoniae*, gut microbiota, immune responses, SCFAs, LAMTOR2, mechanisms

## Abstract

Klebsiella pneumoniae is a common cause of human-pneumonia-derived sepsis with high morbidity and mortality. The microbiota promotes and maintains host immune homeostasis. The mechanisms by which the gut microbiota affects the host defenses in the respiratory system systematically, however, remain poorly understood. Here, we show that gut microbiota depletion increases susceptibility to extracellular K. pneumoniae infections in terms of increased bacterial burdens in lung and decreased survival rates. Oral supplementation with gut microbiota-derived short-chain fatty acids (SCFAs), subsequently activating G protein-coupled receptor 43 (GPCR43), enhances a macrophage’s capacity to phagocytose invading K. pneumoniae. Furthermore, SCFAs and GPR43 increase macrophage bacterial clearance by upregulating LAMTOR2, which is further identified as an antibacterial effector and elucidated to facilitate phagosome-lysosome fusion and extracellular signal-regulated kinase (ERK) phosphorylation. Lastly, conditional ablation of *Lamtor2* in macrophages decreases their antimicrobial activity, even though mice were pretreated with exogenous SCFA supplementation.

**IMPORTANCE** These observations highlight that SCFAs promote macrophage elimination of K. pneumoniae via a LAMTOR2-dependent signal pathway and suggest that it is possible to intervene in K. pneumoniae pneumonia by targeting the gut microbiota.

## Abstract (precis)

These observations highlight that SCFAs promote macrophage elimination of K. pneumoniae via a LAMTOR2-dependent signal pathway and suggest that it is possible to intervene in K. pneumoniae pneumonia by targeting the gut microbiota.

## INTRODUCTION

Klebsiella pneumoniae is a severe multidrug-resistant (MDR) pathogen associated with high morbidity and mortality, which accounts for about one-third of all Gram-negative infections overall, owing to the limited availability of treatment options (1, 2). Being common natural inhabitants of our microbiome, the risk of worldwide spread of these MDR pathogens has become a recognized global threat (2). Therefore, there is an urgent need to expand our understanding of how host defenses limit the pathogenesis and dissemination of K. pneumoniae, and novel therapeutic strategies should be explored.

The past 2 decades witness the implications of gut microbiota in modulating the inflammatory responses both locally and systemically. Several studies demonstrate that alterations in the microbiome and the microbial metabolome are associated with a wide array of diseases (3–5), and concepts of the gut-liver axis, gut-lung axis, and gut-brain axis thus have been proposed, subsequently appreciated and accepted by the academic community. Indeed, the gut-lung axis has already been reported to be associated with respiratory diseases (6–8). Systemic roles of the gut microbiota are attributed mainly to microbiota-derived metabolites, including popularly studied short-chain fatty acids (SCFAs) (9–11). These bacterially derived metabolites are essential elements in the activation of G protein-coupled receptors (GPRs), such as GPR43, GPR41, and OLFR78 (12–15), and in the inhibition of histone deacetylases (HDACs) (16–18). Moreover, SCFAs, especially butyrate, have been demonstrated to promote regulatory T cell homeostasis in the colon (17, 18). Associations between SCFA metabolisms and the development of inflammatory disorders have thus been identified and accepted (19–21). Nevertheless, mechanisms by which SCFAs exert protective effects on distal organs remain controversial, especially in the respiratory system (22, 23).

Numerous pro- or anti-inflammatory mediators have been reported to participate in the elimination of extracellular invading pathogens by macrophages (24–26). LAMTOR2, the late endosomal/lysosomal adaptor mitogen-activated protein kinase (MAPK) and mammalian target of rapamycin (mTOR) activator/regulator complex 2, has been demonstrated to regulate dendritic cell (DC) homeostasis as well as endosomal biogenesis (27–29). In addition, the LAMTOR2 complex is of great significance in correct spatiotemporal extracellular signal-regulated kinase (ERK) phosphorylation and activation (30, 31). Combining multiomic analysis and *in vitro* or *in vivo* assays, we hypothesize that *Lamtor2* deficiency may compromise the ability of macrophages to eliminate pathogenic bacteria.

In this study, by combining mouse models and multiomics analyses, we elucidate explicitly the mechanisms by which the gut microbiota promotes immune responses systematically in the lung during infection by the major human pathogen K. pneumoniae. Our work highlights the physiological functions of both SCFAs and LAMTOR2, demonstrates a heretofore-unrecognized microbiota-SCFA/GPR43-LAMTOR2-pERK-inducible nitric oxide synthase (iNOS) signaling pathway involved in host innate immune responses, and suggests that this pathway may be a novel therapeutic strategy to intervene in K. pneumoniae pneumonia by targeting the gut microbiota.

## RESULTS

### Gut microbiota depletion exacerbates K. pneumoniae-induced lung injury.

**FIG 1:**
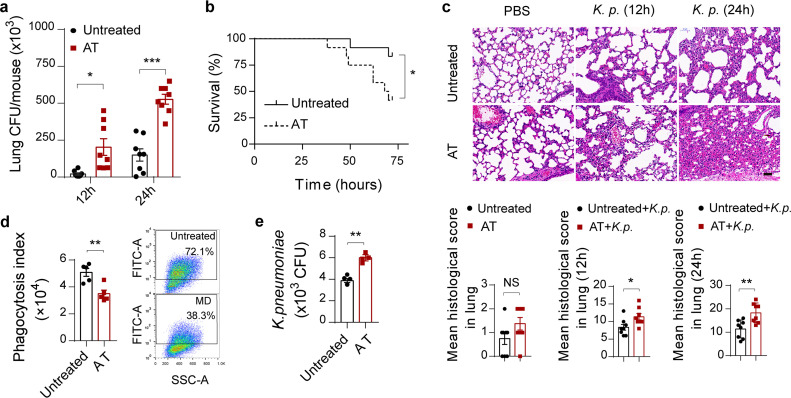
Gut microbiota depletion exacerbates K. pneumoniae-induced lung injury. (a) Pulmonary bacterial burdens in antibiotic-treated and untreated controls 12 h and 24 h after intranasal challenge with 1 × 10^5^ CFU of K. pneumoniae. (b) Survival rates of antibiotic-treated and untreated controls infected by K. pneumoniae. (c) Representative hematoxylin and eosin (H&E) staining and quantification of pathological scores of lung sections derived from antibiotic-treated and untreated controls after K. pneumoniae infection. Scale bars, 50 μm. (d) Phagocytic capacity of isolated alveolar macrophages from antibiotic-treated and untreated controls after K. pneumoniae infection evaluated by flow cytometry (MOI = 100). (e) Bacterial loads of isolated alveolar macrophages from antibiotic-treated and untreated controls. Data are from three independent experiments (a, b, d, e) or one experiment representative of three independent experiments (c) (means ± SEM). The group size was 8 to 12 mice. The *P* values were determined using two-tailed Student *t* tests (a, c to e) or the log rank (Mantel-Cox) test (b). *, *P* < 0.05; **, *P* < 0.01; ***, *P* < 0.001. FITC-A, fluorescein isothiocyanate-area; NS, not significant. AT, antibiotic-treated mice.

To determine the roles of the gut microbiota in regulating antibacterial immunity outside the intestinal lumen, a model of bacterial-infection-induced lung injury was applied. Briefly, we initially treated wild-type mice with broad-spectrum antibiotics in drinking water to deplete gut microbiota, as previously described (8), and then infected them intranasally with 1 × 10^5^ CFU of K. pneumoniae. As with other studies (32, 33), in antibiotic-treated mice, sharp increases in the burdens of K. pneumoniae in the lung (Fig. 1a) and blood (see Fig. S1a in the supplemental material) were observed compared to levels in untreated controls. Pulmonary inflammatory cytokines, such as tumor necrosis factor alpha (TNF-α), interleukin 6 (IL-6), and IL-1β, and chemokines, such as chemokine C-X-C motif ligand 1 (CXCL1) and monocyte chemoattractant protein 1 (MCP-1), were all downregulated in antibiotic-treated mice (Fig. S1b). Bacterial-infection-induced mortality was remarkably elevated in antibiotic-treated mice (Fig. 1b). Taken together, these observations suggested that gut microbiota depletion resulted in compromised defenses against K. pneumoniae, as reflected by increased bacterial burdens in the lung and higher mortality.

10.1128/mSystems.00587-20.1 FIG S1 Protective role of the gut microbiota during K. pneumoniae-induced sepsis. (a) Blood bacterial burdens in antibiotic-treated and untreated controls 12 h and 24 h after K. pneumoniae infection. (b) Pulmonary production of cytokines (TNF-α, 1L-1β, and IL-6) and chemokines (CXCL1 and MCP-1) in antibiotic-treated and untreated controls 24 h after K. pneumoniae infection. (c) Representative H&E staining and quantification of pathological scores of liver and kidney sections from antibiotic-treated and untreated controls 24 h after K. pneumoniae infection. Scale bars, 50 μm. The group size was 8 to 12 mice. Data are from three independent experiments (a, b) or one experiment representative of three independent experiments (c) (means ± SEM). The *P* values were determined using two-tailed Student *t* tests (a to c). *, *P < *0.05; **, *P < *0.01; ***, *P < *0.001. NS, not significant; AT, antibiotic-treated mice. Download FIG S1, TIF file, 2.6 MB. Copyright © 2020 Wu et al. 2020 Wu et al. This content is distributed under the terms of the Creative Commons Attribution 4.0 International license.

To further investigate whether the role of the gut microbiota is protective in the host’s respiratory defense against organ damage triggered by extracellular invading bacteria, we semiquantitatively determined pathology scores in lung, liver, and kidney between antibiotic-treated mice and untreated controls at various time points after K. pneumoniae challenge. Not surprisingly, all infected mice shared histological evidence of severe pneumonia. We found that antibiotic-treated mice displayed earlier and more severe inflammation in the lung (Fig. 1c), liver, and kidney (Fig. S1c). Collectively, these results indicated that the gut microbiota protects against lung injury during K. pneumoniae-induced sepsis.

### Gut microbiota depletion impedes alveolar macrophage function.

The innate immune system acts as the first weapons of invading microorganisms, and residing macrophages in the lung are the key regulators of host innate immunity during bacterial infection and pneumonia (7, 34). Considering the protective effect of the gut microbiota on the host immune defense, we investigated whether microbiota depletion would affect the phagocytosis and clearance of K. pneumoniae by alveolar macrophages. We observed that primary alveolar macrophages originated from antibiotic-treated mice indeed had a markedly diminished capacity to phagocytize and eliminate K. pneumoniae compared to that of controls (Fig. 1f and g). With consideration of the results of other studies (32, 33), we demonstrated that the gut microbiota played protective roles during K. pneumoniae infections.

### Gut microbiota depletion alters profiles of cecum metabolomics and gut microbiomes.

**FIG 2:**
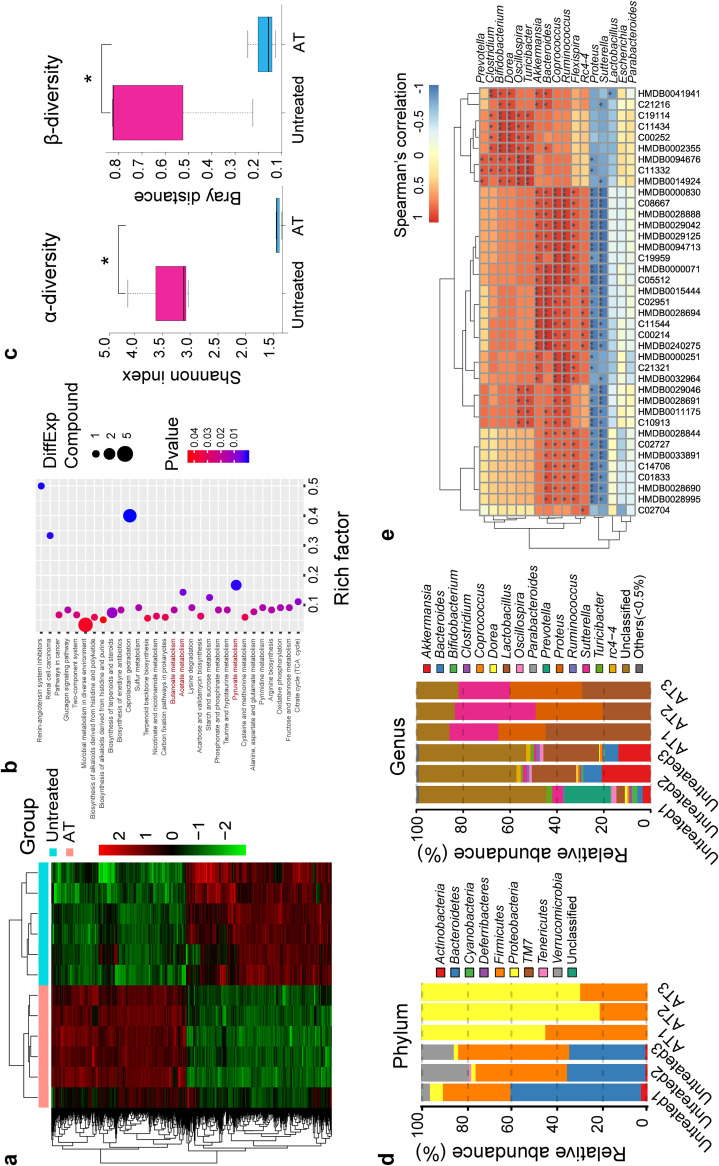
Gut microbiota depletion alters profiles of cecum metabolomics and associated gut microbiomes. (a) Hierarchical clustering heatmap of metabolites identified in cecum contents from antibiotic-treated and untreated controls. Red denotes increased expression; green denotes decreased expression. (b) Selected examples of KEGG pathway enrichment. Disturbances in SCFA metabolism are highlighted in red. (c) α-Diversity (Shannon index) and β-diversity (Bray-Curtis similarity index) of 16S rRNA genes from antibiotic-treated and untreated controls. (d) Discriminative operational taxonomic unit (OTU) abundances of taxonomic distributions at the phylum and genus levels in antibiotic-treated compared to untreated controls. (e) Spearman’s rank correlation between cecum metabolites and associated gut microbiota in antibiotic-treated and untreated controls. Red connections indicate a positive correlation, while blue connections are negative. The *P* values were determined using the hypergeometric test and the Benjamini-Hochberg false-discovery rate (FDR) correction (b) or the two-tailed Wilcoxon rank sum test (e). *, *P* < 0.05; **, *P* < 0.01; ***, *P* < 0.001. AT, antibiotic-treated mice.

As previously described, the way that the microbiota modulates host immunity and susceptibility to infection depends largely on metabolites (35–38). To definitely determine the metabolic profiles of antibiotic-treated mice, we performed in-depth untargeted metabolomics sequencing of cecum samples by liquid chromatography-mass spectrometry (LC-MS). In total, 1,126 metabolites that differed significantly in abundance were identified and isolated in a comparison with controls based on the Kyoto Encyclopedia of Genes and Genomes (KEGG) database (Fig. 2a); these metabolites were associated primarily with carbohydrate and amino acid metabolism. Moreover, we carried out KEGG pathway enrichment analysis (Fig. 2b), which revealed that gut microbiota depletion elicited distinct shifts in metabolic pathways. Consistently with a previous work (39), we observed that SCFA metabolisms were dysregulated (Fig. 2b), which modulates host antimicrobial activity (14, 39, 40). Furthermore, we also analyzed metabolomics profiles of antibiotic-treated and K. pneumoniae*-*infected mice versus K. pneumoniae-infected controls to explore the influences of the gut microbiota on invading extracellular pathogens (Fig. S2a). Discriminations of SCFA metabolisms were also captured (Fig. S2b). Taken together, these results indicated that gut microbiota depletion may give rise to pronounced changes in metabolomics profiles whether specimens are infected with K. pneumoniae or not in a comparison with counterpart control mice.

10.1128/mSystems.00587-20.2 FIG S2 Differential metabolites and KEGG pathway enrichment. (a) Hierarchical clustering heatmap of metabolites identified in cecum contents from K. pneumoniae*-*infected and antibiotic-treated mice versus K. pneumoniae-infected controls. Red denotes increased expression; green denotes decreased expression. (b) Selected examples of KEGG pathway enrichment. Disturbances in propionate and butyrate metabolism are highlighted in red. The *P* values were determined using the hypergeometric test and Benjamini-Hochberg FDR correction (b). AT, antibiotic-treated mice. Download FIG S2, TIF file, 1.3 MB. Copyright © 2020 Wu et al. 2020 Wu et al. This content is distributed under the terms of the Creative Commons Attribution 4.0 International license.

To identify whether the metabolism dysfunction was associated with alterations in the gut microbiomes, we carried out 16S rRNA sequencing on the basis of V4 variable regions. We obtained a total of 736,749 high-quality gene sequences, which were then clustered into operational taxonomic units (OTUs) at a 97% similarity level. As with the previous studies (33), phylogenetic diversity analysis showed remarkable differences in the richness and diversity in antibiotic-treated mice (Fig. 2c), and the compositions of bacterial communities were also altered (Fig. 2d). Notably, the abundances of *Parabacteroides*, *Bifidobacterium*, *Clostridium*, *Coprococcus*, and *Prevotella*, at the genus level, which produced SCFAs, were all decreased in the antibiotic-treated mice (Fig. 2d). Moreover, similar alterations in the bacterial compositions were observed when all mice were infected with K. pneumoniae (Fig. S3a to c). Based on these findings, we proposed that microbiota depletion, whether the host is infected with K. pneumoniae or not, causes a gut microbiome disturbance, with lower abundances of SCFA-producing bacteria. A Spearman correlation analysis revealed an interrelationship between cecum metabolites and the corresponding gut microbiota among gut antibiotic-treated mice and controls infected with K. pneumoniae or not infected (Fig. 2e; Fig. S3d). It was therefore concluded that alteration of metabolites, SCFAs included, may in part be attributed to changes in gut microbial composition and function, subsequently leading to a dysregulation of host innate immunity.

10.1128/mSystems.00587-20.3 FIG S3 Association of cecum metabolites and associated gut microbiomes. (a) α-Diversity (Shannon index) and β-diversity (Bray-Curtis similarity index) of 16S rRNA genes from K. pneumoniae-infected, antibiotic-treated mice and K. pneumoniae-infected controls. (b, c) Discriminative OTU abundances of taxonomic distributions at the phylum (b) and genus (c) levels between K. pneumoniae-infected, antibiotic-treated mice and K. pneumoniae-infected controls. (d) Spearman’s rank correlation between cecum metabolites and associated gut microbiota in K. pneumoniae-infected, antibiotic-treated mice and K. pneumoniae-infected controls. Red connections indicate a positive correlation, while blue connections are negative. The *P* values were determined using a two-tailed Wilcoxon rank sum test (d). *, *P < *0.05; **, *P < *0.01; ***, *P* < 0.001. AT, antibiotic-treated mice. Download FIG S3, TIF file, 2.9 MB. Copyright © 2020 Wu et al. 2020 Wu et al. This content is distributed under the terms of the Creative Commons Attribution 4.0 International license.

### SCFAs enhance antimicrobial activity in macrophages.

**FIG 3:**
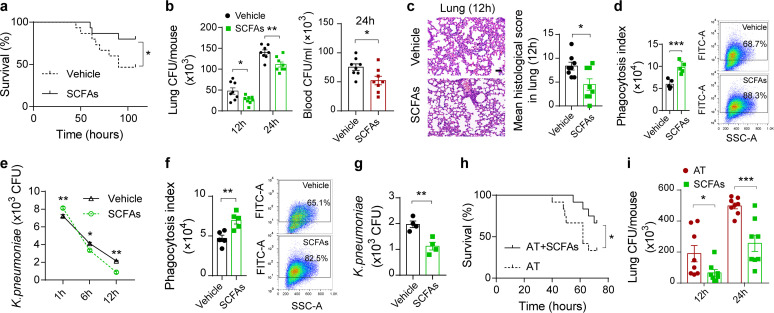
SCFAs promote inflammatory responses during K. pneumoniae-induced sepsis. (a) Survival rates of SCFA-treated mice and untreated controls postinfection with 1 × 10^5^ CFU of K. pneumoniae. (b) Lung and blood bacterial burdens in SCFA-treated mice and untreated controls 12 h and 24 h after K. pneumoniae infection. (c) Representative H&E staining and quantification of pathological scores of lung sections from SCFA-treated mice and untreated controls 12 h after K. pneumoniae infection. Scale bars, 50 μm. (d) Phagocytic capacity of isolated alveolar macrophages from SCFA-treated mice and untreated controls. (e) Bacterial loads of isolated alveolar macrophages from SCFA-treated mice and untreated controls. (f, g) Phagocytosis (f) and clearance (g) of SCFA-treated differentiated THP-1 cells and untreated controls after K. pneumoniae infection. (h) Survival rates of antibiotic-treated mice treated with SCFAs or vehicle. (i) Pulmonary bacterial burdens in antibiotic-treated mice treated with SCFAs or vehicle. The group size was 8 to 12 mice. Data are from three independent experiments (b, d to g, i) or one experiment representative of three independent experiments (a, c, h) (means ± SEM). The *P* values were determined using a log rank (Mantel-Cox) test (a, h) or two-tailed Student *t* tests (b to g, i). *, *P* < 0.05; **, *P* < 0.01; ***, *P* < 0.001.

Recent studies have proposed that the vital link between the microbiota and host immunity is due to the production of SCFAs through bacterial metabolism (41). To assess influences of SCFAs on sepsis triggered by K. pneumoniae, mixtures of SCFAs were administered in drinking water before K. pneumoniae challenge (42). Consistently with the previous studies (14, 39), bacterial-infection-induced mortality was reduced in SCFA-treated mice (Fig. 3a). Furthermore, supplementation with SCFAs significantly reduced the bacterial burdens in the lung and blood (Fig. 3b). Pulmonary pathological scores were improved in SCFA-treated mice (Fig. 3c). Then, we validated whether SCFAs modulated alveolar macrophage physiological function with regard to the phagocytosis and clearance of K. pneumoniae. Comparative analysis showed that continuous exposure to SCFAs contributed to significantly increased capacities of macrophages to internalize and eliminate K. pneumoniae (Fig. 3d and e). Abilities to internalize and clear K. pneumoniae were also enhanced in SCFA-treated differentiated THP-1 cells (Fig. 3f and g). Additionally, we examined whether SCFA supplementation in antibiotic-treated mice could ameliorate immune dysfunction during K. pneumoniae*-*induced sepsis. As a result, supplementation of antibiotic-treated mice with SCFAs led to reduced mortality and decreased bacterial burdens in the lung (Fig. 3h and i). Therefore, we propose that SCFAs enhance host immunity in macrophages during K. pneumoniae infection.

### Gut microbiota depletion alters transcriptome profiles.

**FIG 4:**
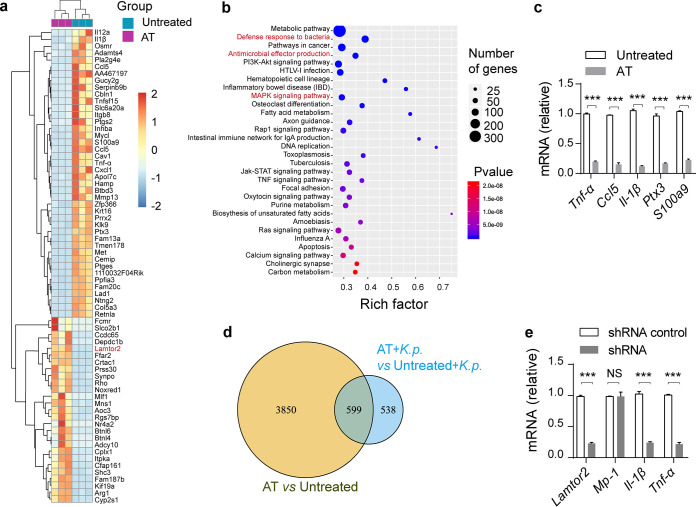
Gut microbiota depletion alters alveolar macrophage transcriptome profiles. (a) Hierarchical clustering heatmap of selected significant differentially expressed genes (DEGs) in alveolar macrophages from antibiotic-treated and untreated controls. Red denotes increased expression; green denotes decreased expression. (b) Selected examples of KEGG pathway enrichment. The defense response to bacteria, antimicrobial effector production, and the MAPK/ROS signaling pathway are highlighted in red. (c) Validation of *Tnf-α*, *Ccl5*, *Il-1β*, *Cxcl1*, and *S100a9* mRNA expression using RT-qPCR. (d) Venn diagram of differentially expressed genes among four groups. (e) Responsiveness of shRNA-mediated *Lamtor2* knockdown RAW264.7 cells and controls in terms of cytokine and chemokine production against K. pneumoniae infection. The group size was 8 to 12 mice. Data are from three independent experiments (c, e). The *P* values were determined using the hypergeometric test and Benjamini-Hochberg FDR correction (b) or two-tailed Student *t* tests (c, e). ***, *P* < 0.001. NS, not significant; AT, antibiotic-treated mice.

To refine our limited understanding of how the gut microbiota exerted its protective effects against invasion by extracellular pathogens, we performed genome-wide transcriptional sequencing of isolated alveolar macrophages and then analyzed gene expression profiles. Consistently with findings from previous literature (8), gut microbiota depletion had a remarkable influence on the alveolar macrophage transcriptome (Fig. 4a). Gene annotation revealed that substantially enriched KEGG pathways were involved in immunity, such as the defense response to bacteria and MAPK/reactive oxygen species (ROS) signaling cascades (Fig. 4b). To validate the RNA sequencing results, quantitative PCR with reverse transcription (RT-qPCR) was conducted. In line with sequencing results, levels of TNF-α, 1L-1β, CCL5, CXCL1, and S100A9 production were all decreased in antibiotic-treated mice (Fig. 4c). Moreover, we also analyzed differences of genome-wide transcriptional signatures between K. pneumoniae*-*infected, antibiotic-treated mice and K. pneumoniae-infected controls (Fig. S4a, b). As with the above experiments, we conducted RT-qPCR assays to confirm the sequencing data (Fig. S4c). To search for commonly different expression genes among the four groups above, we performed a Venn diagram analysis (Fig. 4d). We observed a total of 599 genes, which consisted of 322 downregulated genes and 277 upregulated genes. Then, we tried to screen novel antimicrobial effectors during K. pneumoniae infection of RAW264.7 cells via short hairpin RNA (shRNA)-mediated RNA interference (RNAi). Consistently with the previous study (8), we observed that LAMTOR2 was dysregulated in antibiotic-treated mice, which might be attributed to the gut microbiome. In addition, LAMTOR2 was reported to limit *Salmonella* replications *in vivo* (31). Based on these data, we supposed that LAMTOR2, probably regulated by the gut microbiome, played a significant role in eliminating K. pneumoniae in macrophages. Fortunately, the immune responses were impaired with regard to downregulated proinflammatory effectors when *Lamtor2* expression was knocked down (Fig. S4d and e and Fig. 4e). Taken together, these results suggested that gut microbiota depletion resulted in profound changes in the transcriptional profiles of alveolar macrophages whether K. pneumoniae infection was present or not in a comparison with counterpart controls.

10.1128/mSystems.00587-20.4 FIG S4 Gut microbiota depletion alters transcriptional profiles in infected mice. (a) Hierarchical clustering heatmap of selected significant DEGs between K. pneumoniae-infected, antibiotic-treated mice and K. pneumoniae-infected controls. Red denotes increased expression; green denotes decreased expression. (b) Selected examples of KEGG pathway enrichment. (c) Expression levels of *Tnf-α*, *Il-10*, *Il-1β*, *Mcp-1*, and *Cxcl1* mRNA in K. pneumoniae-infected, antibiotic-treated mice compared to those of K. pneumoniae-infected controls. (d) Immunoblot analysis of LAMTOR2 in shRNA-mediated *Lamtor2* knockdown RAW264.7 cells. (e) Immunoblot confirmation of the LAMTOR2 protein in *Lamtor2*^−/−^ RAW264.7 macrophages. (f) Sequencing of targeted *Lamtor2* mutations and alignment with those from wild-type RAW264.7 cells. Data are from three independent experiments (c) or one experiment representative of three independent experiments (d to f). The *P* values were determined using a hypergeometric test and Benjamini-Hochberg FDR correction (b) or a two-tailed Student *t* test (c). **, *P < *0.01; ***, *P < *0.001. AT, antibiotic-treated mice. Download FIG S4, TIF file, 1.6 MB. Copyright © 2020 Wu et al. 2020 Wu et al. This content is distributed under the terms of the Creative Commons Attribution 4.0 International license.

### LAMTOR2 increases antimicrobial activity in macrophages.

**FIG 5:**
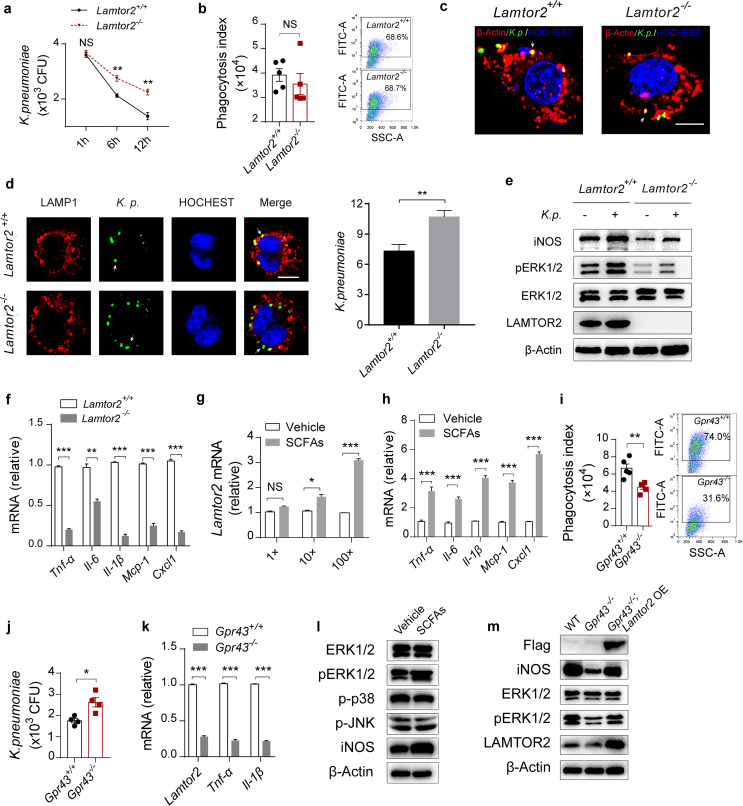
SCFAs facilitate ERK activation by upregulating LAMTOR2. (a, b) Bacterial loads (a) and phagocytic capacity (b) of *Lamtor2*^−/−^ RAW264.7 cells and controls after K. pneumoniae infection. (c) Immunofluorescence analysis of the uptake of K. pneumoniae in *Lamtor2*^−/−^ RAW264.7 cells and controls. Arrows indicate K. pneumoniae. Scale bars, 10 μm. (d, left) Immunofluorescence analysis of colocalization between phagosomes and lysosome. (Right) Count of phagosomes localized with LAMP1-stained lysosome. Arrows indicate K. pneumoniae. Scale bars, 10 μm. (e) Immunoblot analysis of phosphorylated (p-) ERK and iNOS expression levels in *Lamtor2*^−/−^ RAW264.7 cells and controls 6 h after K. pneumoniae infection (MOI = 10). (f) RT-qPCR analysis of *Tnf-α*, *Il-6*, *Il-1β*, *Mcp-1*, and *Cxcl1* mRNA expression in *Lamtor2*^−/−^ RAW264.7 cells and controls 6 h after K. pneumoniae infection (MOI = 10). (g) RT-qPCR analysis of *Lamtor2* mRNA expression in SCFA-treated RAW264.7 cells after K. pneumoniae infection (MOI = 10). (h) Responsiveness of isolated alveolar macrophages from SCFA-treated mice and untreated controls to K. pneumoniae in terms of cytokine and chemokine production *in vitro* (MOI = 10). (i, j) Phagocytosis (i) and clearance (j) of SCFA-treated *Gpr43*^−/−^ RAW264.7 cells and controls. (k) RT-qPCR analysis of *Lamtor2*, *Tnf*-*α*, and *Il-1β* mRNA expression in *Gpr*43^−/−^ RAW264.7 cells and controls, both of which were incubated with SCFAs. (l) Immunoblot analysis of pERK, p-JNK, p-p38, and iNOS expression levels in RAW264.7 cells treated with SCFAs and controls after 6 h of K. pneumoniae infection. (m) Immunoblot analysis of pERK and iNOS in *Gpr43*^−/−^ and *Gpr43*^−/−^ *Lamtor2* overexpression (OE) RAW264.7 cells incubated with SCFAs 6 h after K. pneumoniae infection. The group size was 8 to 12 mice. Data are from three independent experiments (a, b, f to k) or one experiment representative of three independent experiments (c, d, e, l, m) (means ± SEM). The *P* values were determined using two-tailed Student *t* tests (a, b, d, f to k). *, *P* < 0.05; **, *P* < 0.01; ***, *P* < 0.001. NS, not significant; WT, wild type.

To further investigate the role of LAMTOR2 in K. pneumoniae-induced sepsis, we first investigated whether bacterial loads were altered in *Lamtor2*^−/−^ RAW264.7 macrophages (Fig. S4f). As a result, bacterial loads were greater than those from normal controls (Fig. 5a), but there was no difference in the levels of uptake of K. pneumoniae (Fig. 5b and c). Next, we explored mechanisms involved in the LAMTOR2-mediated antibacterial ability of RAW264.7 cells. To start, the absence of *Lamtor2* contributed to a sharp decrease in the count of phagosomes localized with lysosome (LAMP1) (Fig. 5d), which indicated an inappropriate phagosome-lysosome fusion. Additionally, it has been reported that ERK signaling triggers the production of several proinflammatory mediators, thus facilitating the host immune response (43). Consistently with a previous study (31), protein analysis showed noticeable decreases in ERK phosphorylation (pERK) and iNOS expression in *Lamtor2*^−/−^ RAW264.7 macrophages (Fig. 5e), which impeded antimicrobial activity against intracellular pathogens. Cytokine and chemokine production was impaired in *Lamtor2*^−/−^ RAW264.7 cells when they were infected with K. pneumoniae (Fig. 5f). Taken together, these results suggested that LAMTOR2 was necessary for phagosome-lysosome fusion and ERK activation, which collaboratively contributed to bacterial clearance.

### SCFAs facilitate ERK activation by upregulating LAMTOR2.

Next, we explored mechanisms by which SCFAs modulate innate immunity and investigated whether SCFAs collaborate with other inflammatory mediators to potentiate inflammatory and immune responses. Surprisingly, we observed that the expression level of *Lamtor2* was upregulated in a concentration-dependent manner in SCFA-treated RAW264.7 cells 6 h after K. pneumoniae infection (Fig. 5g). As with the *in vitro* results, we found that the *Lamtor2* expression level was elevated *in vivo* when mice were exposed to SCFAs after K. pneumoniae infection (Fig. S5a). Additionally, 6 h after the inoculation of K. pneumoniae *in vitro*, increased expression levels of cytokines (TNF-α, IL-1β, and IL-6) and chemokines (CXCL1 and MCP-1) were also observed compared to levels in controls without SCFA supplementation (Fig. 5h).

10.1128/mSystems.00587-20.5 FIG S5 Determination of *Lamtor2* mRNA expression *in vivo* and generation of *Gpr43*^−/−^ RAW264.7 cells. (a) RT-qPCR analysis of *Lamtor2* mRNA expression in isolated alveolar macrophages after K. pneumoniae infection. The *P* values were determined using two-tailed Student *t* tests. ***, *P* < 0.001. (b) Immunoblot validation of GPR43 in *Gpr43*^−/−^ RAW264.7 cells. (c) Sequencing of targeted *Gpr43* mutations in *Gpr43*^−/−^ RAW264.7 cells and alignment with those from wild-type controls. Data are from three independent experiments (a) or one experiment representative of three independent experiments (b, c). Download FIG S5, TIF file, 0.8 MB. Copyright © 2020 Wu et al. 2020 Wu et al. This content is distributed under the terms of the Creative Commons Attribution 4.0 International license.

It has been reported that GPR43/FFAR2 plays an important role in the gut-lung axis, serving as a sensor of host microbiota activity (14). Using *Gpr43*^−/−^ RAW264.7 macrophages (Fig. S5b, c), we observed that the phagocytosis index was decreased compared to that of controls (Fig. 5i), and bacterial loads were tremendously increased (Fig. 5j). On the basis of these results, we proposed that SCFAs promoted K. pneumoniae uptake and clearance in a GPR43-dependent manner in macrophages. RT-qPCR assay showed that the expression level of *Lamtor2* was decreased significantly in *Gpr43*^−/−^ cells when they were treated with SCFAs and incubated with K. pneumoniae for 6 h, with the production of TNF-α and IL-1β significantly reduced (Fig. 5k). As previously depicted, SCFAs modulated MAPK signaling cascades in a GPR43-dependent manner in human renal cortical epithelial cells (HRCEs) (44). We further explored whether SCFAs/GPR43 had impacts on LAMTOR2 signaling in stimulating inflammatory responses. We investigated the protein levels of pERK, p-JNK, and p-p38 in alveolar macrophages when mice were exposed to SCFAs and then K. pneumoniae. The expression level of pERK was increased, with p-JNK and p-p38 exhibiting no observed alterations (Fig. 5l). We then overexpressed LAMTOR2 in normal and *Gpr43*^−/−^ RAW264.7 macrophages (Fig. S6a). As expected, the production of inflammatory cytokines (TNF-α, IL-1β, and IL-6) and chemokines (CXCL1 and MCP-1) was elevated (Fig. S6b). Moreover, LAMTOR2 overexpression in *Gpr43*^−/−^ RAW264.7 cells partly rescued ERK activation (Fig. 5m) and restored their ability to clear a K. pneumoniae infection (Fig. S6c). Therefore, we concluded that SCFA/GPR43 propagated phagocytosis in a LAMTOR2-independent manner, while promoting elimination of a K. pneumoniae infection in a LAMTOR2-dependent manner (Fig. 3d and Fig. 5b).

10.1128/mSystems.00587-20.6 FIG S6 LAMTOR2 overexpression in RAW264.7 cells. (a) Immunoblot analysis of LAMTOR2 protein in LAMTOR2-overexpressed RAW264.7 cells. (b) Expression levels of *Tnf-α*, *Il-6*, *Il-1β*, *Mcp-1*, and *Cxcl1* mRNA in LAMTOR2-overexpressed RAW264.7 cells after K. pneumoniae infection. (c) Bacterial loads of *Gpr43*^−/−^ and *Gpr43*^−/−^ *Lamtor2* overexpression (OE) in RAW264.7 cells. Data are from three independent experiments (b, c) or one experiment representative of three independent experiments (a). The *P* values were determined using two-tailed Student *t* tests (b, c). **, *P < *0.01; ***, *P < *0.001. Download FIG S6, TIF file, 1.0 MB. Copyright © 2020 Wu et al. 2020 Wu et al. This content is distributed under the terms of the Creative Commons Attribution 4.0 International license.

### *Lamtor2* conditional disruption attenuates antimicrobial activity *in vivo*.

**FIG 6:**
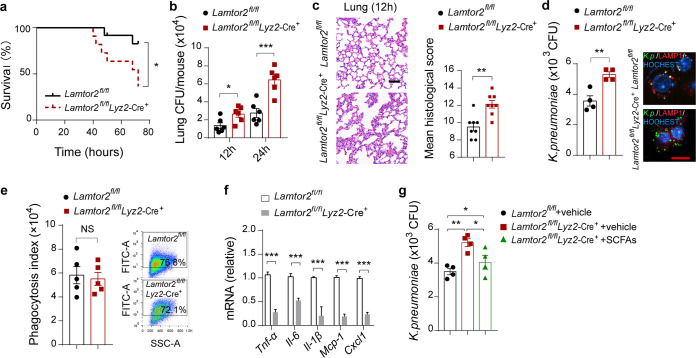
*Lamtor2* loss of function leads to a decreased antimicrobial activity *in vivo*. (a) Survival rates of *Lamtor2^fl^*^/^*^fl^ Lyz2*-Cre^+^ and littermate *Lamtor2^fl^*^/^*^fl^* mice intranasally infected with K. pneumoniae. (b) Pulmonary bacterial burdens of *Lamtor2^fl^*^/^*^fl^ Lyz2*-Cre^+^ mice and controls 12 h and 24 h after K. pneumoniae infection. (c) Representative H&E staining and quantification of pathological scores of lung sections from *Lamtor2^fl^*^/^*^fl^ Lyz2*-Cre^+^ mice and controls 12 h after K. pneumoniae infection. Scale bars, 50 μm. (d) Bacterial loads of isolated alveolar macrophages from *Lamtor2^fl^*^/^*^fl^ Lyz2*-Cre^+^ mice and controls after K. pneumoniae infection. Scale bars, 20 μm. (e) Phagocytic capacity of isolated alveolar macrophages from *Lamtor2^fl^*^/^*^fl^ Lyz2*-Cre^+^ mice and controls after K. pneumoniae infection. (f) Responsiveness of isolated alveolar macrophages from *Lamtor2^fl^*^/^*^fl^Lyz2*-Cre^+^ mice and controls to K. pneumoniae in terms of TNF-α, IL-6, 1L-1β, MCP-1, and CXCL1 production. (g) Bacterial loads of isolated alveolar macrophages from *Lamtor2^fl^*^/^*^fl^ Lyz2*-Cre^+^ mice and controls treated with SCFAs after K. pneumoniae infection. The group size was 8 to 12 mice. Data are from three independent experiments (b, d to g) or one experiment representative of three independent experiments (a, c) (means ± SEM). The *P* values were determined using a log rank (Mantel-Cox) test (a), two-tailed Student *t* tests (b to f), or one-way ANOVA for multiple comparisons (g). *, *P < *0.05; **, *P < *0.01; ***, *P < *0.001.

To investigate whether the functions of LAMTOR2 on macrophage activation are relevant *in vivo*, we constructed a *Lamtor2* conditional knockout mouse using CRISPR/Cas9-mediated genome editing and crossed the mouse with *Lyz2*-Cre^+^ transgenic mice to generate *Lamtor2^fl^*^/^*^fl^ Lyz2*-Cre^+^ mice (Fig. S7a to d). We observed that *Lamtor2^fl^*^/^*^fl^ Lyz2*-Cre^+^ mice had an elevated mortality after K. pneumoniae infection compared to that of littermate *Lamtor2^fl^*^/^*^fl^* controls (Fig. 6a). In line with observations *in vitro*, bacterial burdens in the lungs and blood of *Lamtor2^fl^*^/^*^fl^ Lyz2*-Cre^+^ mice were tremendously increased 12 h and 24 h after K. pneumoniae infection (Fig. 6b and Fig. S7e). In addition, we analyzed the pulmonary pathology of *Lamtor2^fl^*^/^*^fl^ Lyz2*-Cre^+^ and littermate controls 12 h and 24 h after K. pneumoniae challenge. Consequently, an exacerbated lung pathology with enhanced interstitial inflammation, bronchitis, and large surfaces of confluent inflammation infiltration was observed (Fig. 6c and Fig. S7f). Moreover, bacterial loads were evaluated in isolated alveolar macrophages from *Lamtor2^fl^*^/^*^fl^ Lyz2*-Cre^+^ mice and compared to those from *Lamtor2^fl^*^/^*^fl^* mice (Fig. 6d); the phagocytosis indexes were also evaluated and determined to have no alteration (Fig. 6e). In addition, the responsiveness of isolated primary alveolar macrophages derived from *Lamtor2^fl^*^/^*^fl^ Lyz2*-Cre^+^ mice were decreased compared to those of *Lamtor2^fl^*^/^*^fl^* mice in response to invading K. pneumoniae (Fig. 6f) and lipopolysaccharide (LPS) (Fig. S7g), respectively.

10.1128/mSystems.00587-20.7 FIG S7 Generation of *Lamtor2* conditional knockout (cKO) mice via CRISPR-Cas9 genome editing. (a) Schematic generation of *Lamtor2* conditional knockout mice. Targeted exons 1 to 3 of the *Lamtor2* gene were flanked by Loxp sites. Maps of the *Lamtor2* wild-type allele, targeting vector, *Lamtor2*-targeted allele, and *Lamtor2* knockout allele are shown. (b) Genotyping of wild-type (*Lamtor2*^+/+^), fl/+ (*Lamtor2^fl^*^/+^), and fl/fl (*Lamtor2^fl^*^/^*^fl^*) mice was performed by PCR. (c) RT-qPCR analysis of *Lamtor2* mRNA expression in targeted alveolar macrophages. (d) Immunoblot analysis of the LAMTOR2 protein in targeted alveolar macrophages. (e) Blood bacterial burdens in *Lamtor2^fl^*^/^*^fl^ Lyz2*-Cre^+^ and *Lamtor2^fl^*^/^*^fl^* mice 12 h and 24 h after K. pneumoniae infection. (f) Representative H&E staining and quantification of pathological scores of lung sections from *Lamtor2^fl^*^/^*^fl^ Lyz2*-Cre^+^ mice and controls 24 h after K. pneumoniae infection. Scale bars, 50 μm. (g) Responsiveness of isolated alveolar macrophages from *Lamtor2^fl^*^/^*^fl^* Lyz2-Cre^+^ mice and controls against lipopolysaccharide (LPS) in terms of TNF-α, IL-6, 1L-1β, MCP-1, and CXCL1 production. Data are from three independent experiments (c, e, g) or one experiment representative of three independent experiments (b, d, f) (means ± SEM). The *P* values were determined using two-tailed Student *t* tests (c, e, f, g). **, *P < *0.01; ***, *P < *0.001. Download FIG S7, TIF file, 1.6 MB. Copyright © 2020 Wu et al. 2020 Wu et al. This content is distributed under the terms of the Creative Commons Attribution 4.0 International license.

Next, we investigated whether isolated alveolar macrophages derived from *Lamtor2^fl^*^/^*^fl^ Lyz2*-Cre^+^ mice treated with SCFAs exhibited an increased ability to eliminate extracellular invading bacteria compared to counterparts without SCFAs. Clearly, macrophages from *Lamtor2* mutant mice treated with SCFAs harbored fewer K. pneumoniae organisms 6 h postinfection than untreated *Lamtor2* mutant mice but more than treated *Lamtor2^fl^*^/^*^fl^* controls (Fig. 6g). Accordingly, these results indicated that SCFAs were associated with the LAMTOR2 signaling cascades in modulating antimicrobial activity *in vivo*.

## DISCUSSION

Pneumonia remains a leading cause of death and hospitalization worldwide, especially among children and the elderly (1, 2). Early and aggressive antibiotic treatments indeed exerted great control over these bacterial infections (45). Recent breakthroughs in our understanding of the protective role of the gut microbiota in our health have novel implications for respiratory and critical care medicine. In this study, we showed that the gut microbiota played a protective role in K. pneumoniae-induced lung impairment and highlighted that SCFAs enhanced macrophage phagocytosis of K. pneumoniae independently of LAMTOR2, while promoting host elimination of infected K. pneumoniae via a LAMTOR2-dependent signal pathway. We take a new step toward understanding the relationship between the gut microbiota and remote organs or immune cells with respect to the inflammatory response to extracellular invading pathogens.

It has become apparent that large communities of intestinal microbes not only fine-tune immune cell function locally in the vicinity of the mucosa but also exert a systemic influence on effector cells of the innate immune system at extraintestinal tissues or organs (46, 47). The mechanistic basis for these distal influences, however, has been incompletely characterized. In the lung, it has been suggested that resistance to multiple bacterial and viral pathogens is enhanced by the gut microbiota (8, 33). Trompette et al. once established the concept of the gut-lung axis in allergic airway disease by showing that gut microbiota-derived metabolites have an influence on the severity of allergic inflammation (48). Recently, Clarke demonstrated that gut microbiota depletion causes significant defects in the early innate response to lung infection by the major human pathogen K. pneumoniae and proposed that nod-like receptor ligands are required to facilitate early bacterial clearance from the lung (32). Consistently with these results, we show that the gut microbiota protects against lung injury during K. pneumoniae-induced sepsis, resulting in decreased bacterial burdens and higher survival rates.

Recent advances have identified SCFAs as an “indispensable linker” in host microbiota communication networks. Initially described as a fuel resource for epithelial cells (49), hepatocytes and peripheral tissues are now rapidly emerging as critical signals that directly influence immunity and cell function (42, 50). Prime mechanisms involved in SCFA immunomodulatory effects in neutrophils and macrophages are through binding and activating GPCRs. Indubitably, both *Gpr41*^−/−^ and G*pr43*^−/−^ mice had reduced immune responses to Citrobacter rodentium infection (15). Work conducted by Galvão et al. suggested that the microbial metabolic sensor GPR43 modulated the lung’s innate immunity to bacterial pneumonia through binding ligand acetate (14). The cascades of SCFAs involved in regulating immune responses, including downstream targets, however, need to be further considered. In the present study, we show for the first time that SCFAs/GPR43 promote inflammatory responses against K. pneumoniae by activating the intracellular antibacterial effector LAMTOR2. LAMTOR2 initially was reported to combine with MP-1 to modulate MAPK signaling within a cell (27). Afterwards, LAMTOR2 was identified as a mediator in the immune response, as described for patients harboring a LAMTOR2 point mutation allele and, as a consequence, suffering from recurrent bronchopulmonary infections (51). Among the literature are observations that mice with LAMTOR2-deficient dendritic cells have a severe disturbance of the DC compartment (29). In the present report, we show that *Lamtor2*^−/−^ RAW264.7 cells have increased bacterial loads, which were ascribed to inappropriate transport of phagosomes to lysosomes and impaired ERK activation. Furthermore, conditional ablation of *Lamtor2 in vivo* resulted in K. pneumoniae populating excessively where alveolar macrophage antimicrobial responses were not appropriately activated.

Our work highlights the concept that the antibacterial activity of alveolar macrophages in the distal lung is programmed and corrected systemically by signals derived from the gut microbiota. Nevertheless, whether there are other novel gut microbiota-derived factors and metabolites contributing to these effects remains to be further investigated. In addition, how these gut microbial metabolite SCFAs in combination with the host internal antibacterial effector LAMTOR2 regulate the host immune system needs to be further elucidated. More importantly, it remains to be determined whether the observed effects of gut microbiota depletion on K. pneumoniae infection also apply to infections with other important causative agents of pneumonia. On the other hand, characterizing microbial biomarkers has enormous potential for precision medicine and is a relatively simple way of translating microbiome research into clinical practice. In this report, we underlined for the first time the influence of gut microbiota depletion together with *K. pneumonia* challenge of the alveolar macrophage transcriptome and cecum metabolism, which were correlated with obviously observed phenotypes accompanied by a significantly diminished capacity to phagocytize and eliminate K. pneumoniae. Of clinical significance, thus, is how altered alveolar macrophage transcriptome and gut microbial metabolites are associated with K. pneumoniae pneumonia. Based on these data, therefore, it may be worthwhile exploring future new diagnostic biomarkers and novel therapeutics in bacterial pneumonia treatment targeting the gut microbiota.

## MATERIALS AND METHODS

### Mice.

Wild-type C57BL/6 mice were purchased from the Experimental Animal Center of Anhui Province (Hefei, China). *Lamtor2^fl^*^/+^ mice (C57BL/6J) were generated by CRISPR-Cas9-mediated genome-editing technology (Cyagen Biosciences, China) targeting exons 1 to 3. *Lamtor2* conditional knockout mice were generated by crossing *Lamtor2^fl^*^/^*^fl^* mice with *Lyz2*-Cr_e_^+/−^ transgenic mice. Meanwhile, we obtained *Lamtor2^fl/fl^* littermate controls. All experiments involving mice were approved by the Institutional Animal Care and Use Committee of Anhui Medical University (Hefei, China).

### Bacterial strains and cells.

K. pneumoniae (ATCC 43816) was cultured in Luria broth (LB) at 37°C overnight. HEK293T, RAW264.7, and THP-1 cell lines were all obtained from the American Type Culture Collection (ATCC) and cultured in endotoxin-free Dulbecco’s modified Eagle’s medium (DMEM) containing 10% fetal bovine serum (FBS; Gibco) and 1% penicillin/streptomycin (Thermo Fisher, USA). Moreover, THP-1 monocytes were incubated with 100 ng/ml phorbol myristate acetate (PMA) for at least 48 h to differentiate into macrophages.

### Reagents and consumable material.

Main reagents and consumable material are listed in Table S1 in the supplemental material. Antibodies are listed in Table S2.

10.1128/mSystems.00587-20.8 TABLE S1 Reagents and materials. Download Table S1, PDF file, 0.02 MB. Copyright © 2020 Wu et al. 2020 Wu et al. This content is distributed under the terms of the Creative Commons Attribution 4.0 International license.

10.1128/mSystems.00587-20.9 TABLE S2 Antibodies. Download Table S2, PDF file, 0.1 MB. Copyright © 2020 Wu et al. 2020 Wu et al. This content is distributed under the terms of the Creative Commons Attribution 4.0 International license.

### Models of microbiota depletion and K. pneumoniae infection.

Mice were treated with broad-spectrum antibiotics (ampicillin, 1 g · liter^−1^; neomycin sulfate, 1 g · liter^−1^; metronidazole, 1 g · liter^−1^; and vancomycin, 0.5 g · liter^−1^) administered in drinking water for 14 days (8, 33). Antibiotic treatment was stopped 3 days prior to infection. Then, antibiotic-treated mice and untreated controls were anesthetized with pelltobarbitalum natricum, inoculated intranasally with 1 × 10^5^ CFU of K. pneumoniae in 50 μl of sterile phosphate-buffered saline (PBS), and sacrificed at different time points postinfection. Pathology scoring was conducted as described in reference 8; the total lung inflammation score was counted as the sum of the scores for each parameter, the maximum being 24, and the maximum score for liver and kidney sections was 12. To quantify bacterial burdens in lung, mice were sacrificed, and then organs were removed, homogenized in PBS, and cultivated on LB agar plates. For survival curves, mice were monitored 4 to 5 times daily until mice with characteristics of reduced movement, shivering, dyspnea, or circling were killed.

### Isolation of alveolar macrophages.

Alveolar macrophages were isolated as previously described (52). Briefly, mice were sacrificed and immediately exsanguinated. Bronchoalveolar lavage fluid (BALF) was collected with 4 ml of 37°C sterile PBS containing 0.5 mM EDTA. Cells were pelleted and resuspended in RPMI 1640 supplemented with 5.0% (vol/vol) FBS and then were allowed to adhere to a tissue culture flask for 2 h (37°C, 5% CO_2_ [vol/vol]). In general, alveolar macrophage purity was more than 93% as analyzed by flow cytometry (FACS Celesta; BD Biosciences).

### Phagocytosis and killing assays.

Bacterial phagocytosis and killing assays were performed essentially as described previously (8, 46). To begin, a suspension of bacteria was suspended in PBS (pH 9.0) and marked with carboxyfluorescein succinimidyl ester (CFSE; Invitrogen, the Netherlands), with stirring at 37°C for 30 min. Meanwhile, RAW264.7 cells, differentiated THP-1, or alveolar macrophages were incubated in complete medium without antibiotics for at least 2 h and then infected with K. pneumoniae (multiplicity of infection [MOI] = 100). After 1 h of incubation, cells were washed with complete medium without antibiotics but supplemented with 0.05% gentamicin (50 g · liter^−1^) and then were washed with cold PBS (pH 7.3). Subsequently, cells were analyzed by flow cytometry (FACS Celesta; BD Biosciences) for phagocytosis (the phagocytosis index is the geometric mean fluorescence times the percentage of positive cells) or incubated for the other times indicated (3 h, 6 h, and 12 h) and then lysed for killing assays. Briefly, cells were lysed with 0.3% (vol/vol) Triton X-100 for 5 min. Cell lysates were then serially diluted with PBS and inoculated on LB agar plates. Bacterial CFU were counted after incubation of the specimens at 37°C for 16 h.

### Generation and validation of stable gene knockout cell lines.

To generate stable gene knockout cell lines, 5-μg plasmids encoding single guide RNAs (sgRNAs) were transfected using Lipofectamine 2000 (Invitrogen), with strict adherence to the manufacturer´s instructions. Puromycin selection was performed for 1 day at 1 mg · liter^−1^; cells were then split into a 96-well plate (Corning, USA), with 2 to 3 cells/well, and clones were picked 10 days later. Clones were expanded into 6-well dishes, and validation was performed by Western blotting and sequencing.

### SCFA mixture treatment assay.

As conducted previously (14, 42), groups of 8 or 10 wild-type mice were pretreated with SCFA mixtures administered in drinking water (100 mM acetate, 25 mM propionate, and 25 mM butyrate) (Sigma-Aldrich) for 7 days (with fresh solutions three or four times a week). Then, mice were anesthetized with isoflurane and inoculated intranasally with 1 × 10^5^ CFU of K. pneumoniae in 50 μl PBS. In addition, SCFA mixtures were added to RAW264.7 cells *in vitro* for 24 h at concentrations of 100× in the peripheral blood of healthy people (20 mM acetate, 0.5 mM propionate, and 0.5 mM butyrate). Then, RAW264.7 cells were infected with K. pneumoniae at an MOI of 1:100.

### Immunofluorescence assay.

Quantification of bacterial killing and LAMTOR2-associated phagolysosome maturation were determined with a Zeiss LSM-800 confocal microscope (Carl Zeiss, Germany). Briefly, *Lamtor2*^−/−^ RAW264.7 cells and wild-type controls (1 × 10^5^ cells/well) were seeded in 4-well chamber slides (ThermoFisher Scientific, catalog [cat.] no. 155383) and then were infected with CSFE-K. pneumoniae (MOI = 100) for 1 h. Cells were next treated with gentamicin (100 g · liter^−1^) in incomplete medium to kill extracellular K. pneumoniae. Next, cells were immediately fixed on ice for 15 min with 4% paraformaldehyde after being washed twice with cold PBS containing gentamicin and then permeabilized with 0.5% TritonX-100 for 30 min at room temperature. Fixed and permeabilized cells were blocked with 5% bovine serum albumin in PBST (0.5% Tween 20 in PBS) for 30 min, incubated for 2 h with the primary antibody, and then washed three times with PBST and incubated for 60 min with the secondary antibody. Additionally, cells were stained with Hoechst 33258 (Sigma) to clarify the boundaries of the nucleus. Finally, images of macrophages were taken as z-stacks of multiple sections collected at 0.5-mm intervals at ×63 magnification with a Zeiss 800 inverted confocal microscope (Carl Zeiss, Germany). Data were processed with a Light Cycler 96 SW 1.1 (Roche, Germany).

### DNA extraction and 16S rRNA sequencing.

Mouse fecal samples were collected before the mice were sacrificed, frozen immediately following collection, and stored at –80°C prior to analysis. Fecal samples were pulverized with a mortar and pestle in liquid nitrogen, and bacterial genomic DNA was then extracted with a QIAamp DNA stool minikit (Qiagen). V4 region amplicon sequencing (515F-GTGCCAGCMGCCGCGGTAA and 806R-GGACTACHVGGGTWTCTAAT) (53) of the 16S rRNA gene was performed on an Illumina HiSeq2500 sequencer at the Beijing Genomics Institute (BGI-Shenzhen, China). mothur (http://www.mothur.org/) (54) was used to obtain unique reads. Sequences of less than 200 bp and greater than 1,000 bp as well as sequences containing any primer mismatches, barcode mismatches, ambiguous bases, and homopolymer runs exceeding 6 bases were all excluded. All remaining sequences were assigned to operational taxonomic units (OTUs) with a 97% threshold of pairwise identity and then classified taxonomically using the RDP database (http://www.mothur.org/wiki/RDP_reference_files) (55). These taxonomies were used to construct summaries of the taxonomic distributions of OTUs, which can then be applied to calculate the relative abundances of microbiota at different levels.

### RNA extraction, reverse transcription, quantitative real-time PCR, and RNA sequencing.

RNA was extracted from RAW264.7 cells or alveolar macrophages homogenates using an RNeasy Plus minikit (Qiagen, cat. no. 74134) by strictly following the manufacturer’s protocol. For reverse transcription, single-strand cDNA was synthesized using a PrimeScript 1st-strand cDNA synthesis kit (TaKaRa, cat. no. D6110A). Real-time PCR was performed using PrimeScript RT master mix (TaKaRa, cat. no. RR036A) by a three-step real-time PCR system (Light Cycler 96). The target gene expression levels were normalized to that of the housekeeping gene (*Gapdh*) mRNA, determined by the 2^–ΔΔ^*^CT^* calculation method, where *CT* is the threshold cycle. Primers for real-time PCR are listed in Table S3. RNA sequencing was performed by the Beijing Genomics Institute (BGI-Shenzhen, China) using the BGISEQ-500 platform. The sequencing libraries were constructed as described in a previous study (56). Expression levels for each of the genes were normalized to the number of fragments per kilobase of the exon model per million mapped reads (FPKM) by RNA sequencing expectation maximization (RSEM). Differentially expressed genes (DEGs) were analyzed as indicated at http://david.abcc.ncifcrf.gov (57). Pathways enriched with DEGs were annotated in the KEGG database (Kyoto Encyclopedia of Genes and Genomes) (58).

10.1128/mSystems.00587-20.10 TABLE S3 Oligonucleotides. Download Table S3, PDF file, 0.1 MB. Copyright © 2020 Wu et al. 2020 Wu et al. This content is distributed under the terms of the Creative Commons Attribution 4.0 International license.

### Cecal content metabolic sequencing.

Cecal contents were subsequently extracted at the death of the mice and then frozen immediately at –80°C. For metabolomics profiling, all cecum samples were thawed on ice, and a quality control (QC) sample, made by mixing and blending equal volumes (10 μl) of each cecum sample, was used to estimate a mean profile representing all the analytes encountered during analysis. We isolated and extracted metabolites (<1,500 Da) as follows. First, 100-μl cecum mixtures were precipitated with 200 μl methanol, and similarly, the QC sample was precipitated with methanol (1:2, vol/vol). All samples were subsequently centrifuged at 14,000 × *g* for 10 min at 4°C. The supernatants were subjected to metabolomics profiling by liquid chromatography mass spectrometry (LC-MS) at the Beijing Genomics Institute (BGI-Shenzhen, China). The acquired MS data pretreatments, including peak selection and grouping, retention time correction, a second peak grouping, and isotope and adduct annotation, were performed as previously described (59). LC-MS raw data files were converted into the mzXML format and then analyzed by the XCMS and CAMERA toolbox with the R statistical language (v3.4.1). The online KEGG database (http://www.genome.jp/kegg/) (58) and HMDB database (http://www.hmdb.ca) (60) were used to identify different metabolites. If a mass difference between observed and theoretical masses was less than 10 ppm, the metabolite name was reported and the molecular formulas of the matched metabolites were further validated by isotopic distribution measurements. Commercial reference standards were used to validate and confirm metabolites by comparison of their retention times and MS/MS spectra.

### Flow cytometry.

For RAW246.7 cells or alveolar macrophages, flow cytometry was conducted as follows. Briefly, 500 μl of cold 50 mM EDTA in PBS was added to each well and incubated for at least 30 min at 37°C in 5% CO_2_ in a cell culture incubator. Subsequently, the cells were transferred to FACS tubes and centrifuged at 1,000 × *g* for 10 min. The supernatant was carefully removed, and the cell pellet was resuspended in 200 μl of freshly prepared staining solution. The samples were incubated in the dark for 25 min, and subsequently 200 μl of FACS buffer (2 mM EDTA in 10% PBS) was added until the sample was analyzed by a FACSCalibur flow cytometer (FACS Celesta, BD Biosciences). Electronic compensation was used to eliminate bleed-through fluorescence.

### Statistical analysis.

All results are presented as means ± standard errors of the means (SEM). Statistical analysis was performed using unpaired Student *t* tests for two groups and one-way analysis of variance (ANOVA) or two-way ANOVA for multiple groups, with all data points showing a normal distribution. Mouse survival data were plotted as Kaplan-Meier curves and compared using the log rank (Mantel-Cox) test. Sample sizes were selected on the basis of preliminary results to ensure an adequate power. The study and experiments were not randomized or carried out in a blind manner. The results were considered statistically significant or very significant when *P* values were less than 0.05 or 0.01, respectively. All graphs were generated using Adobe Illustrator CC (2017 release) or GraphPad Prism 7.

### Data availability.

Sequencing data and relevant files have been uploaded to public repositories. LC-MS data were deposited into the China National GenBank Database with the accession no. CNP0001290. The 16S sequencing data are available in GenBank with the accession no. MW011789 to MW012255. Transcriptome sequencing data can be found on the NCBI Sequence Read Archive (SRA) with the accession no. PRJNA662812.
