# Supplementary figures and images for: Microbiota-Derived Short-Chain Fatty Acids Promote LAMTOR2-Mediated Immune Responses in Macrophages
Source: mSystems. 2020 Nov 3;5(6):e00587-20. doi: 10.1128/mSystems.00587-20 (PMC7646525; doi:10.1128/mSystems.00587-20)

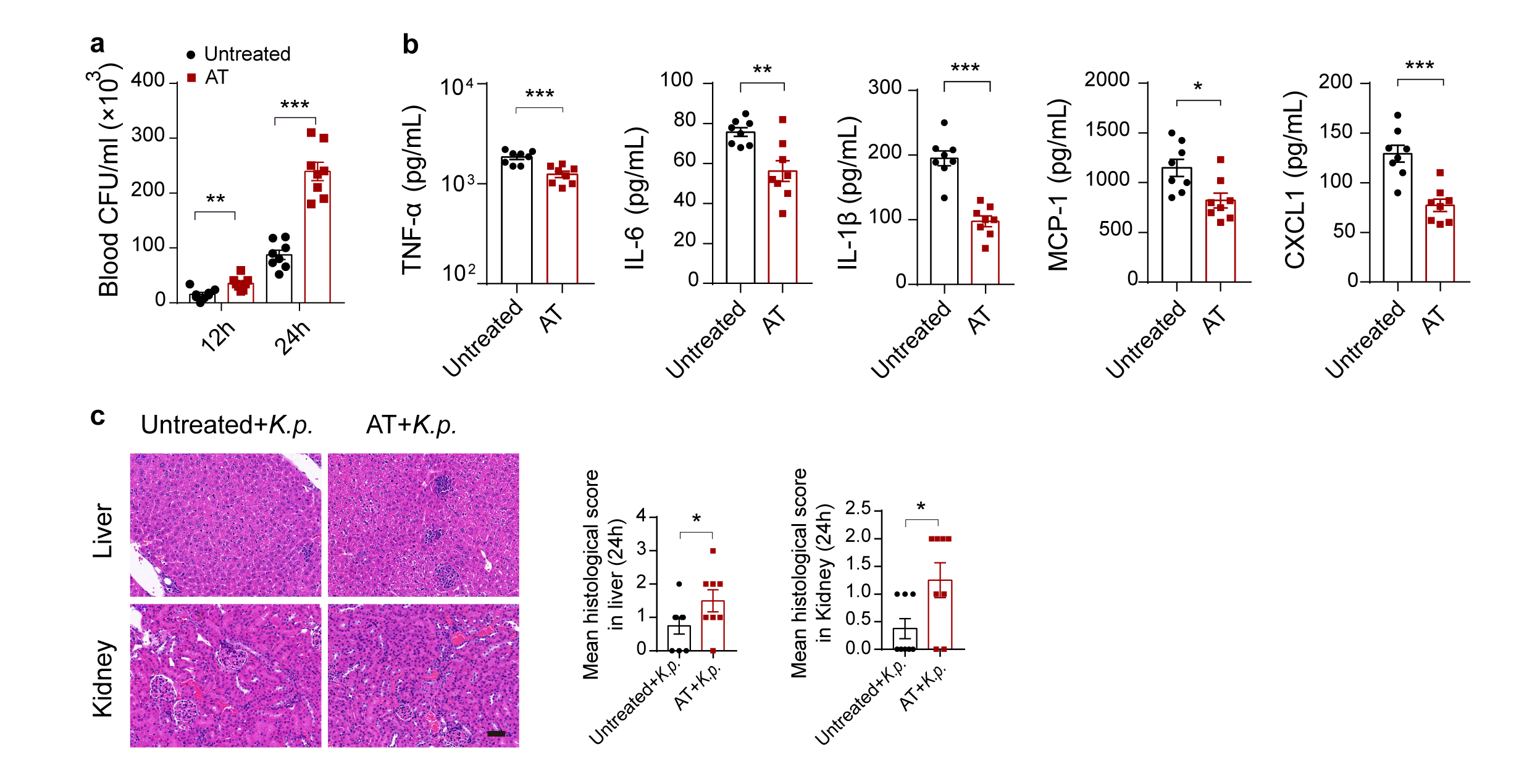

Supplement: FIG S1 [file mSystems.00587-20-sf001.tif]

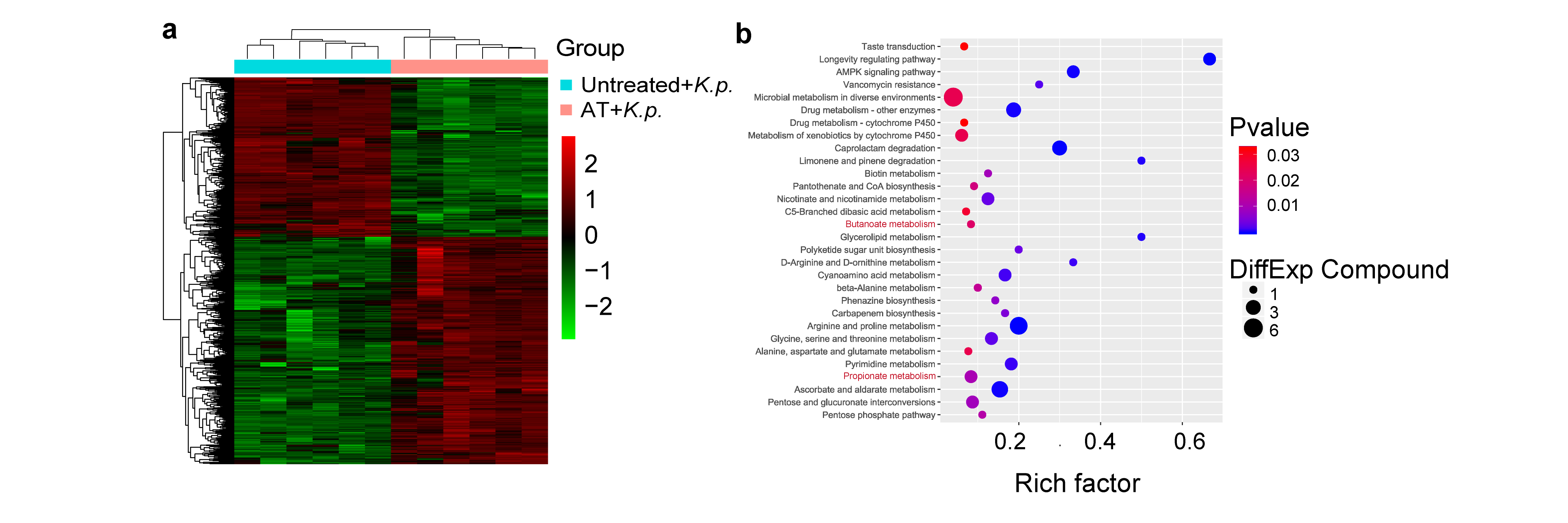

Supplement: FIG S2 [file mSystems.00587-20-sf002.tif]

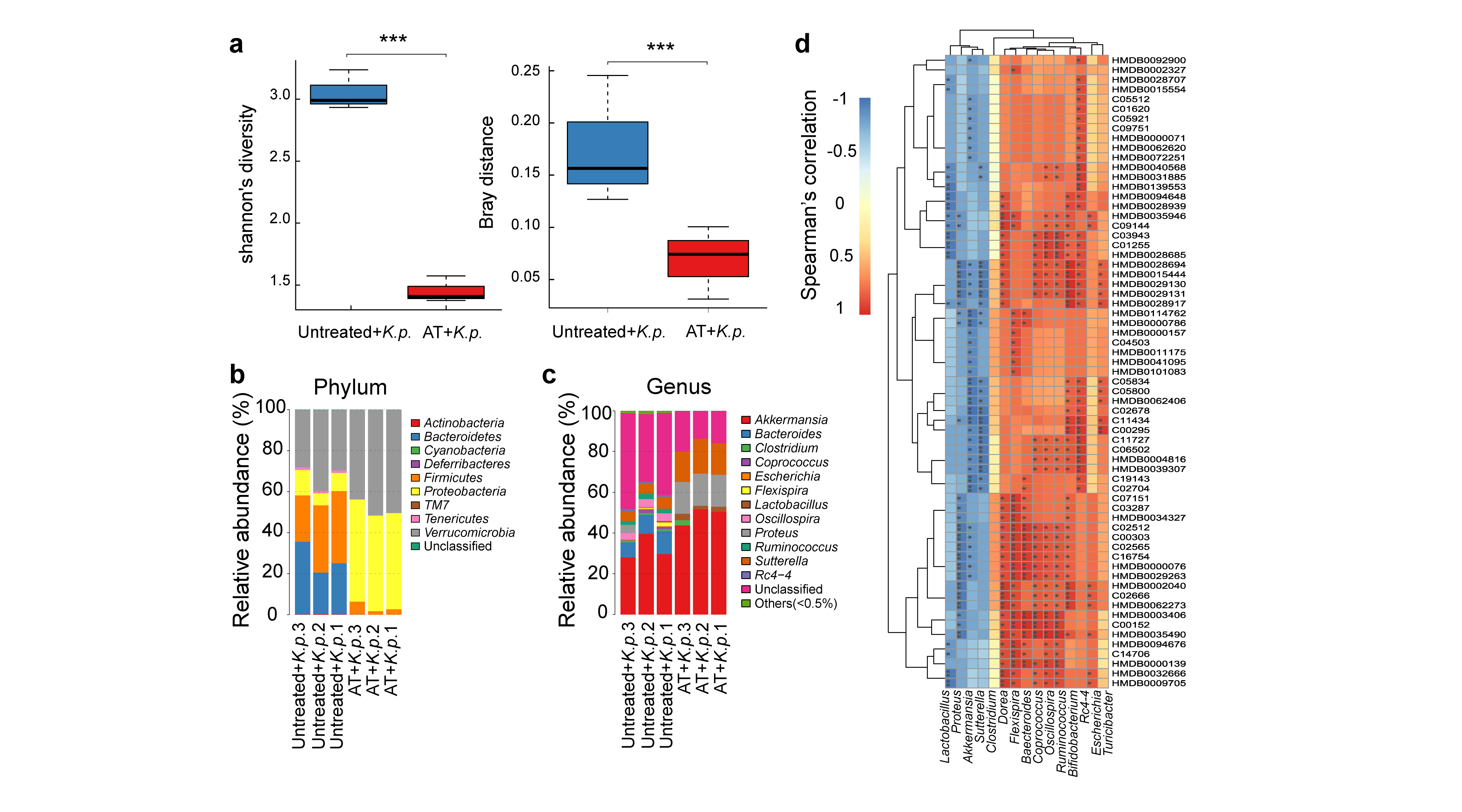

Supplement: FIG S3 [file mSystems.00587-20-sf003.tif]

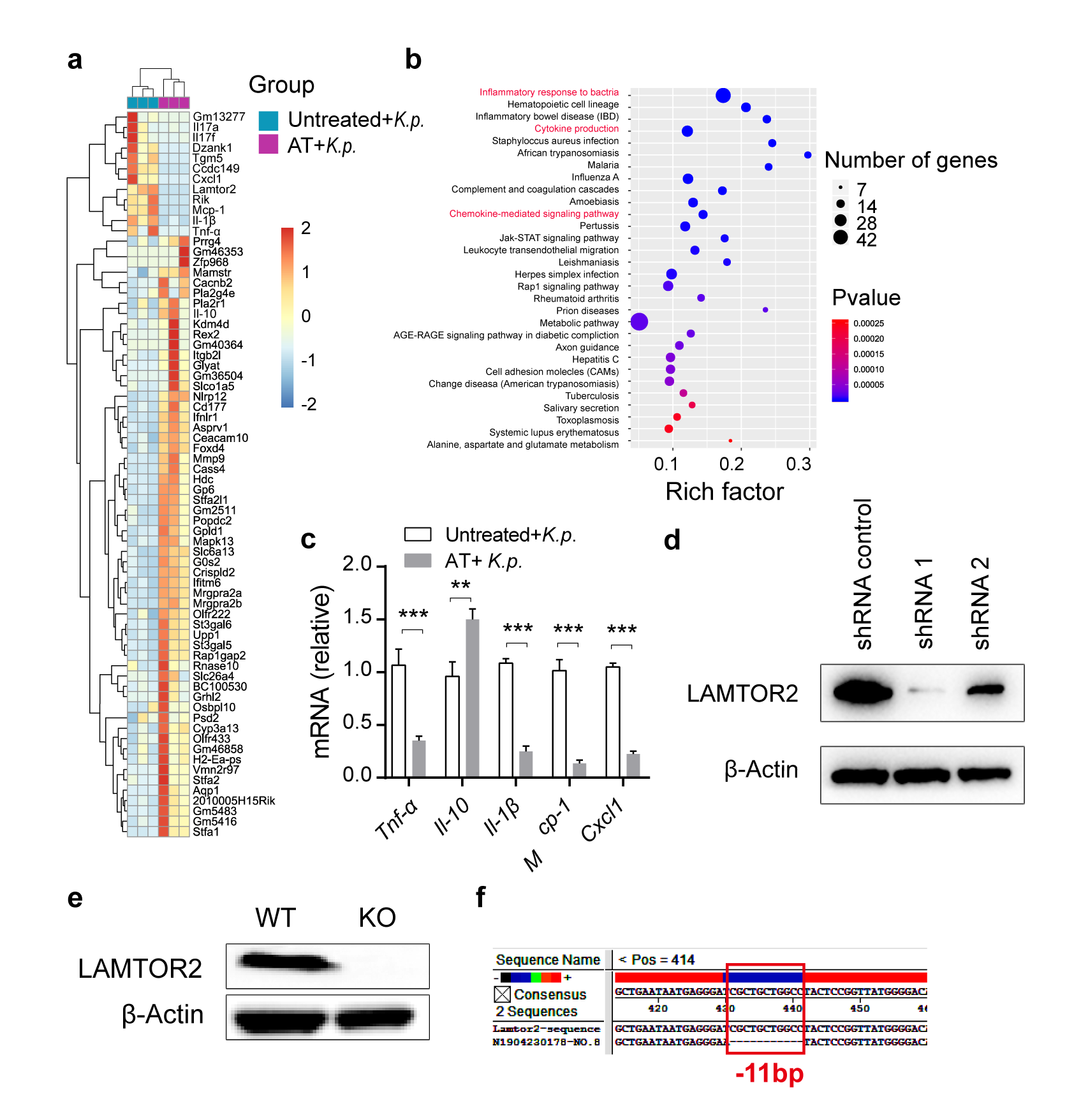

Supplement: FIG S4 [file mSystems.00587-20-sf004.tif]

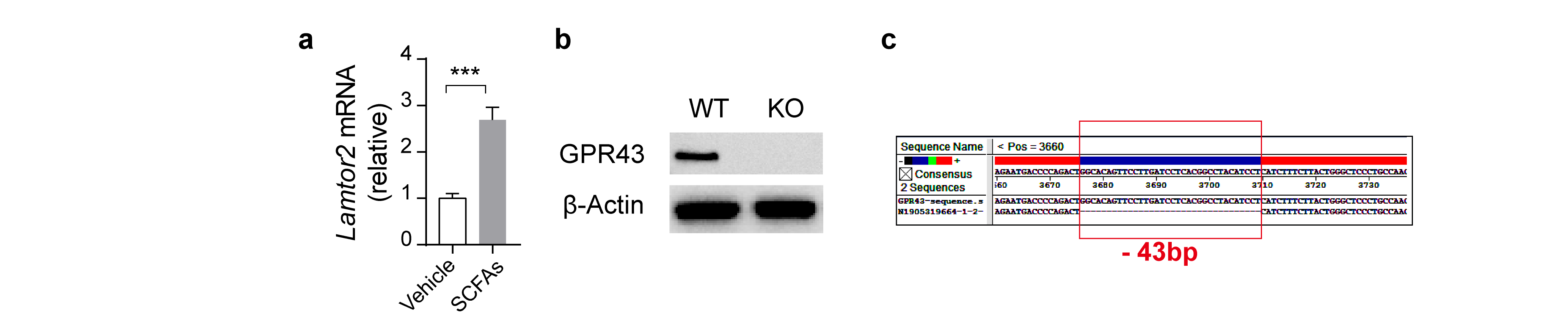

Supplement: FIG S5 [file mSystems.00587-20-sf005.tif]

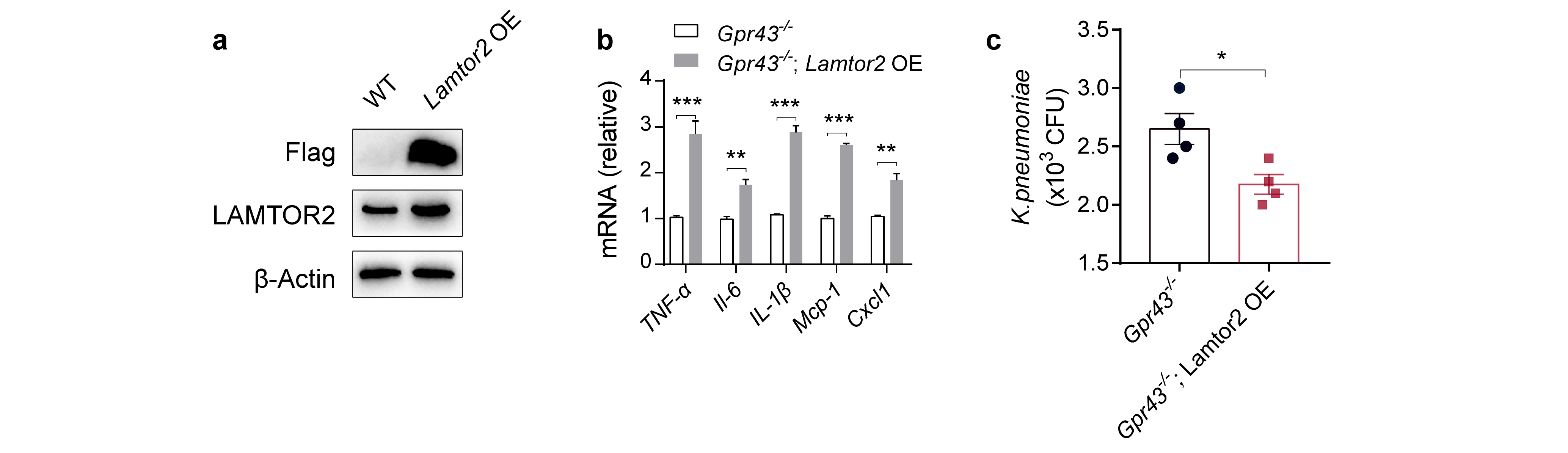

Supplement: FIG S6 [file mSystems.00587-20-sf006.tif]

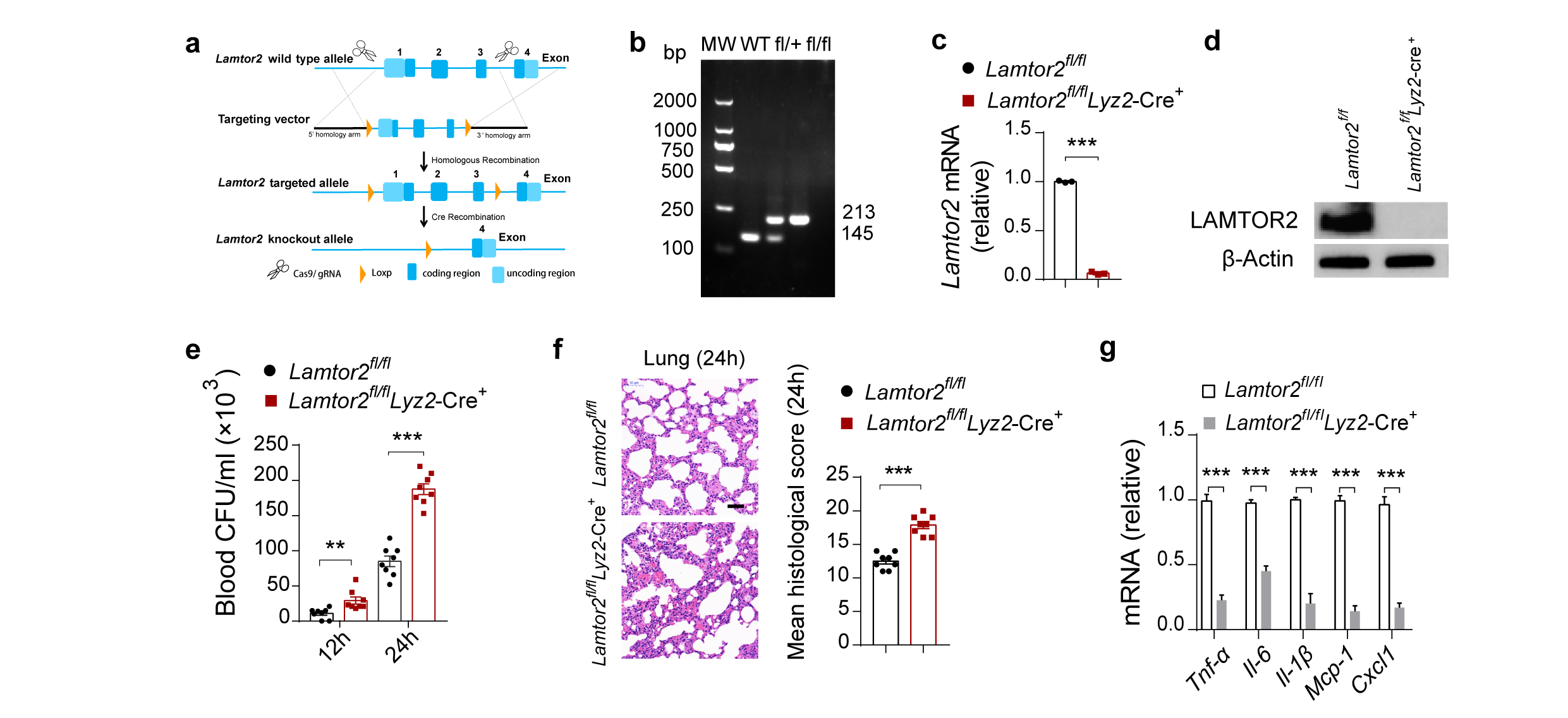

Supplement: FIG S7 [file mSystems.00587-20-sf007.tif]
