# Supplementary material for: Microbiota-Derived Short-Chain Fatty Acids Promote LAMTOR2-Mediated Immune Responses in Macrophages
Source: mSystems. 2020 Nov 3;5(6):e00587-20. doi: 10.1128/mSystems.00587-20 (PMC7646525; doi:10.1128/mSystems.00587-20)
Supplement: TABLE S1 [file mSystems.00587-20-st001.pdf]

Name	Company	Catalog
DMEM	Gibico	Catalog# 11965-092
MEM	Gibico	Catalog# 31985070
RPMI 1640 Medium	Gibico	Catalog# 31870082
Fetal Bovine Serum	Gibico	Catalog# 16000-044
Sodium Butyrate	Sigma Aldrich	Catalog# 303410-100G
Sodium Acetate	Sigma Aldrich	Catalog# S2889-250G
Sodium Propionate	Sigma Aldrich	Catalog# P1880-100G
Penicillin/streptomycin	Sigma Aldrich	Catalog# P4333-100ML
Gentamicin	Sigma Aldrich	Catalog# G1397-10ML
Ampicillin	Sigma Aldrich	Catalog# A9518-25G
Neomycin sulfate	Sigma Aldrich	Catalog# N6386-100G
Metronidazole	Sigma Aldrich	Catalog# M3761-25G
Puromycin	Invitrogen	Catalog# A1113802
CFSE	Invitrogen	Catalog# 65-0850-84
Lipofectamin2000	Invitrogen	Catalog# 11668027
Hoechst 33258	Sigma	Catalog# 861405
Protease Inhibitor Cocktail	Roche	Catalog# 4693116001
TRIzol™ Reagent	Invitrogen	Catalog# 15596018
Lipopolysaccharide (LPS)	Sigma Aldrich	Catalog# L2630-10MG
