# Supplementary material for: Microbiota-Derived Short-Chain Fatty Acids Promote LAMTOR2-Mediated Immune Responses in Macrophages
Source: mSystems. 2020 Nov 3;5(6):e00587-20. doi: 10.1128/mSystems.00587-20 (PMC7646525; doi:10.1128/mSystems.00587-20)
Supplement: TABLE S2 [file mSystems.00587-20-st002.pdf]

**Antibodies**	**Company**	**Catalog**
Anti-β-actin	Santa Cruz Biotechnology	Catalog# sc-47778
Anti-GPR43	Santa Cruz Biotechnology	Catalog# sc-293202
Anti-LAMTOR2	Cell Signaling Technology	Catalog# 8145
Anti-p-p44/42 MAPK(Erk1/2）	Cell Signaling Technology	Catalog# 4370
Anti-p44/42 MAPK(Erk1/2)	Cell Signaling Technology	Catalog# 4695
Anti-LAMP-1	Santa Cruz Biotechnology	Catalog# sc-20011
Anti-p-JNK	Cell Signaling Technology	Catalog# 9255S
Anti-p-p38	Cell Signaling Technology	Catalog# 4511T
Anti-F4/80	Abcam	Catalog# ab111101
Anti-iNOS antibody	Abcam	Catalog# ab15323
Anti-β-Tubulin Antibody	Cell Signaling Technology	Catalog# 2146
Anti-CD14	BioLegend	Catalog# 301804
Anti-CD11b/c	Abcam	Catalog# ab1211
Anti-Flag	Proteintech	Catalog# 66008-3-lg
Goat anti-Mouse (HRP)	Abcam	Catalog# ab6808
Goat Anti-Rabbit (HRP)	Abcam	Catalog# ab97051
Mouse Inflammation Kit	BD bioscience	Catalog# 552364
Goat anti-Mouse, Fluor 555	Invitrogen	Catalog# A32727
Goat anti-Mouse, Fluor 488	Invitrogen	Catalog# A-11001
Goat anti-Rabbit, Fluor 488	Invitrogen	Catalog# A-11008
