# Supplementary material for: Microbiota-Derived Short-Chain Fatty Acids Promote LAMTOR2-Mediated Immune Responses in Macrophages
Source: mSystems. 2020 Nov 3;5(6):e00587-20. doi: 10.1128/mSystems.00587-20 (PMC7646525; doi:10.1128/mSystems.00587-20)
Supplement: TABLE S3 [file mSystems.00587-20-st003.pdf]

Oligonucleotides	5'→3'
Lamtor2 gRNA	GAATAATGAGGGATCGCTGCTGG
Gpr43 gRNA	GCACAGTTCCTTGATCCTCACGG
Lamtor2-genomic-Fw	GTGGCTAATGTTGCAAGCTG
Lamtor2-genomic-Re	ATACTTGCAAAGTCTGCTCGG
Gpr43-genomic-Fw	TGCAGCTTAATATCCCGCCC
Gpr43-genomic-Re	ATCCACACGAAGCGCCAATA
Gapdh-Fw	GTCAAGGCCGAGAATGGGAA
Gapdh-Re	CTCGTGGTTCACACCCATCA
TNFα-Fw	ACTGAACTTCGGGGTGATCG
TNFα-Re	TTGAGATCCATGCCGTTGGC
i-NOS-Fw	AAGATGGCCTGGAGGAATGC
i-NOS-Re	CGTACCGGATGAGCTGTGAA
1L-1β-Fw	CATCCAGCTTCAAATCTCGCA
1L-1β-Re	GATGAAGGAAAAGAAGGTGCTC
1L-6-Fw	ACTTCACAAGTCGGAGGCTTA
1L-6-Re	ATCCAGTTTGGTAGCATCCATC
LAMTOR2-Fw	TGCTAAGCCAAGCCAACACT
LAMTOR2-Re	GCCTACTTTACTCCATACCATGC
MP-1-Fw	CAACAGACCAAGGCAGCAAAC
MP-1-Re	GCTAGCTCCTTTTCTAGGCTGAC
S100A9-PF	AGCATAACCACCATCATCGACA
